# NMR study of a soluble Guanylate Cyclase (sGC) human homologue: the H-NOX domain from Nostoc sp.

**DOI:** 10.1186/2050-6511-16-S1-A73

**Published:** 2015-09-02

**Authors:** Aikaterini Argyriou, Marina Bantzi, Athanassios Giannis, Andreas Papapetropoulos, Georgios A Spyroulias

**Affiliations:** 1 Department of Pharmacy, University of Patras, GR-26504, Patras, Greece; 2 Institute of Organic Chemistry, Leipzig University, D-04103 Leipzig, Germany; 3 School of Health Sciences, Faculty of Pharmacy, University of Athens, GR-15 771, Athens, Greece

## 

Heme-nitric oxide/oxygen binding (H-NOX) motifs can be found as proteins of approximately 200 amino acids in length or can exist as a domain within larger proteins, such as soluble guanylate cyclase. The H-NOX domain is conserved across eukaryotes and bacteria; within sGC, the H-NOX domain functions as a sensor for the gaseous signaling agent nitric oxide (NO). Soluble guanylate cyclase (sGC) contains a heme-binding N-terminal domain that regulates the catalytic site contained within the C-terminal end of the enzyme. sGC is a heterodimer, consisting of α1 or α2 subunit bound to β1 and catalyzes the conversion of GTP to GMP. Activation of NO by sGC increases its activity several hundred-fold, promoting vasodilation and inhibiting platelet aggregation. Under pathophysiological conditions characterized by oxidative stress, sGC suffers heme loss, becomes unresponsive to NO and is tagged for degradation by the ubiquitin-proteasome pathway, leading to compromised NO signaling and cardiovascular disease. Ligands, such as BAY 58-2667, activate sGC in a heme-independent manner and protect heme-oxidized sGC from proteasome degradation. Herein, we present a preliminary NMR investigation of the conformational and electronic properties of the heme-bound H-NOX protein from Nostoc sp., which shares a 35% sequence identity with the H-NOX domain of human sGC. Additionally, we use UV-visible and heteronuclear NMR spectroscopy in order to investigate the structural integrity, the conformational variations and the dynamics of the H-NOX polypeptide during oxidation of the Fe(II) ion, while data on the changes/destabilization of the heme moiety upon the addition of a number of ligands and oxidizing agents (NO, BAY 58-2667, ODQ) are acquired through NMR. Monitoring the dynamical behavior of the H-NOX domain and the alterations occurring in its structure triggered by the changes in the oxidation status of the Fe(II)-Protporphyrin IX prosthetic group in solution by NMR, may provide valuable insights for sGC activation/stimulation and NO signaling.

